# Supplementary material for: Pain from Internal Organs and Headache: The Challenge of Comorbidity
Source: Diagnostics (Basel). 2024 Aug 12;14(16):1750. doi: 10.3390/diagnostics14161750 (PMC11354044; doi:10.3390/diagnostics14161750)
Supplement: Supplementary file 1 [file diagnostics-14-01750-s001.zip › Table S1.pdf]

MAIN VISCERAL PAIN – HEADACHE COMORBIDITIES. TABLE S1.

A. THORACIC INTERNAL ORGANS		
	Heart	CAD + Migraine
B. ABDOMINAL INTERNAL ORGANS		
	Intestine	IBS + Migraine / TTH
	Gallbladder	Gallbladder disease + Migraine
C. PELVIC INTERNAL ORGANS		
	Female reproductive organs	i) Primary Dysmenorrhea + Migraine
		ii) Dysmenorrhea secondary to Endometriosis +Migraine
		iii) Chronic pelvic pain + Migraine
	Urinary bladder	PBS + Migraine

CAD = coronary artery disease; IBS = irritable bowel syndrome; TTH; tension-type headache; PBS: painful bladder syndrome;
