# Supplementary material for: Pain from Internal Organs and Headache: The Challenge of Comorbidity
Source: Diagnostics (Basel). 2024 Aug 12;14(16):1750. doi: 10.3390/diagnostics14161750 (PMC11354044; doi:10.3390/diagnostics14161750)
Supplement: Supplementary file 1 [file diagnostics-14-01750-s001.zip › Table S2.pdf]

MAIN MECHANISMS OF VISCERAL PAIN – HEADACHE COMORBIDITIES. TABLE S2.

CAD + Migraine	Pro-inflammatory substances, endothelial dysfuncion, oxidative stress, dyslipidemia, genetic factors, hormonal asset
IBS + Migraine / TTH	Genetic predisposition, neuroimmunity, neuroendocrine factors, central sensitization
Gallbladder disease + Migraine	Central sensitization
Primary Dysmenorrhea + Migraine	Prostaglandin hyperproduction<br>Central sensitization
Dysmenorrhea secondary to Endometriosis +Migraine	Genetic factors, hormonal factors, central sensitization
Chronic pelvic pain + Migraine	Central sensitization
PBS + Migraine	Central sensitization

CAD = coronary artery disease; TTH = tension-type headache ; IBS = irritable bowel syndrome; PBS = painful bladder syndrome;
